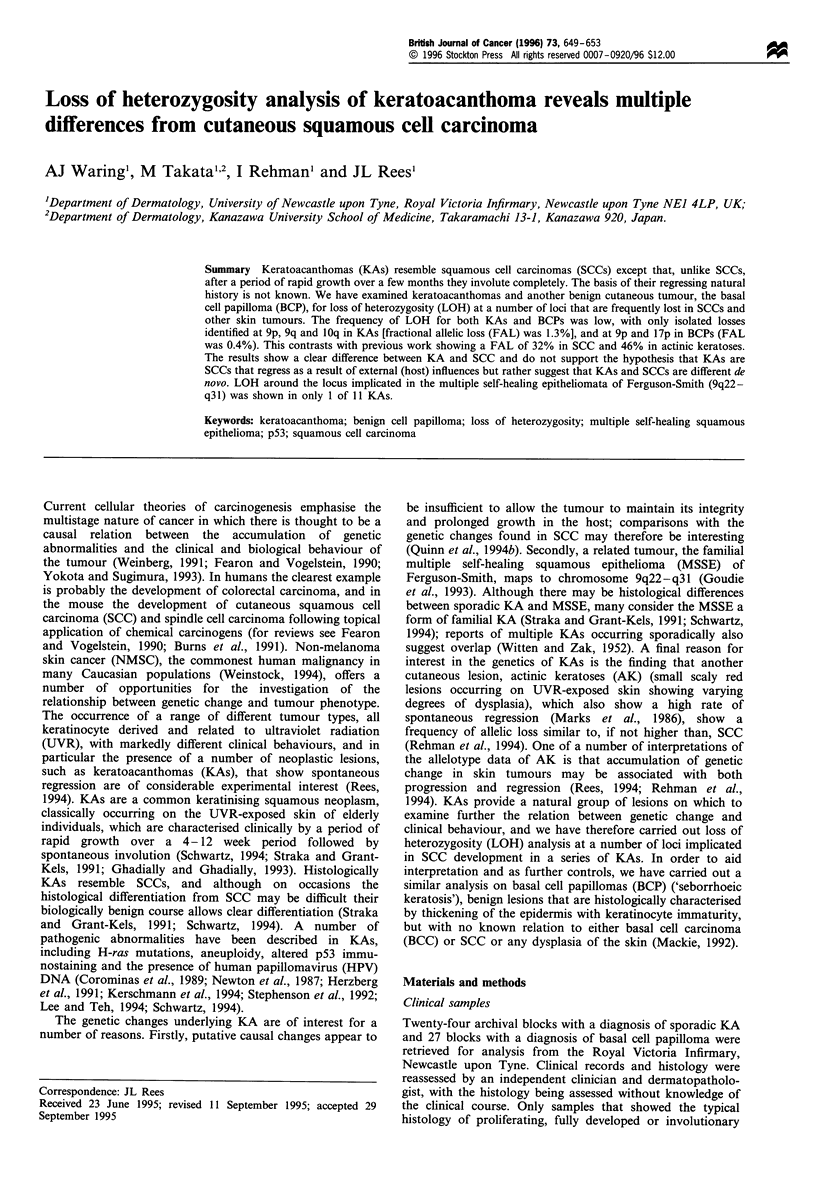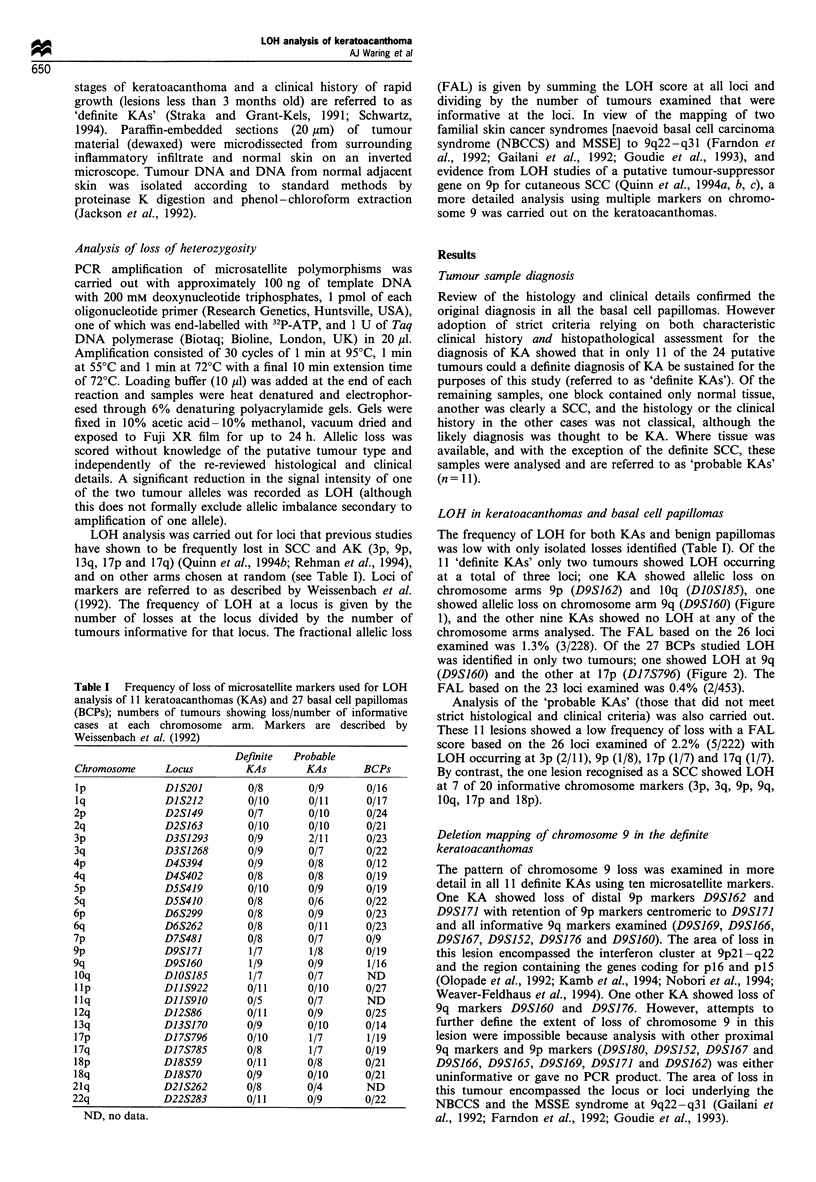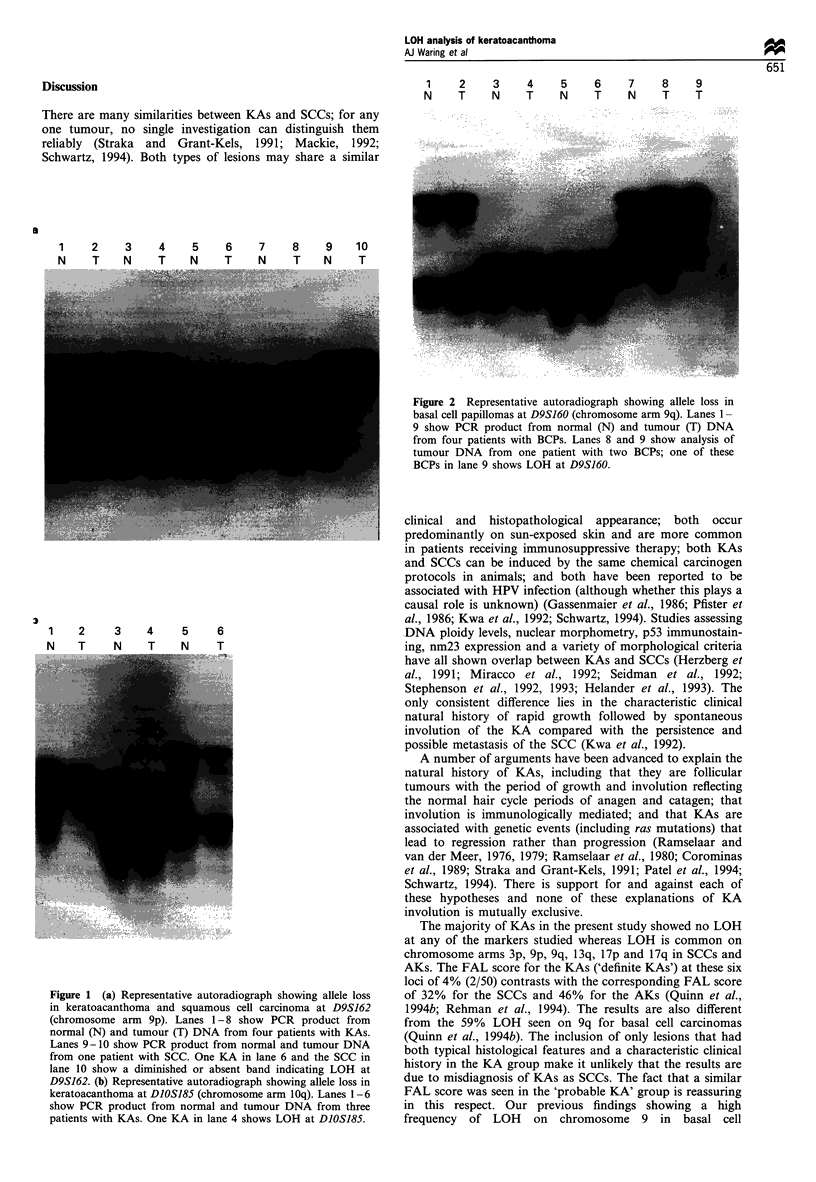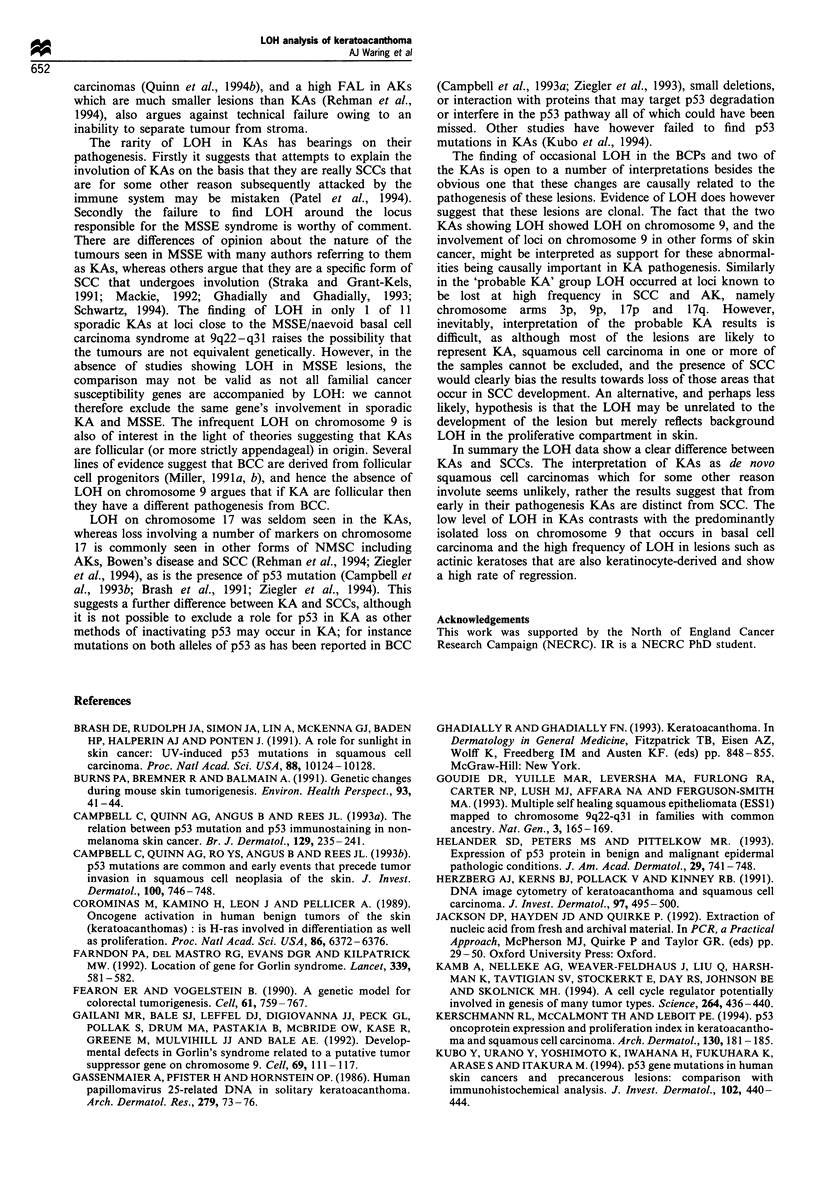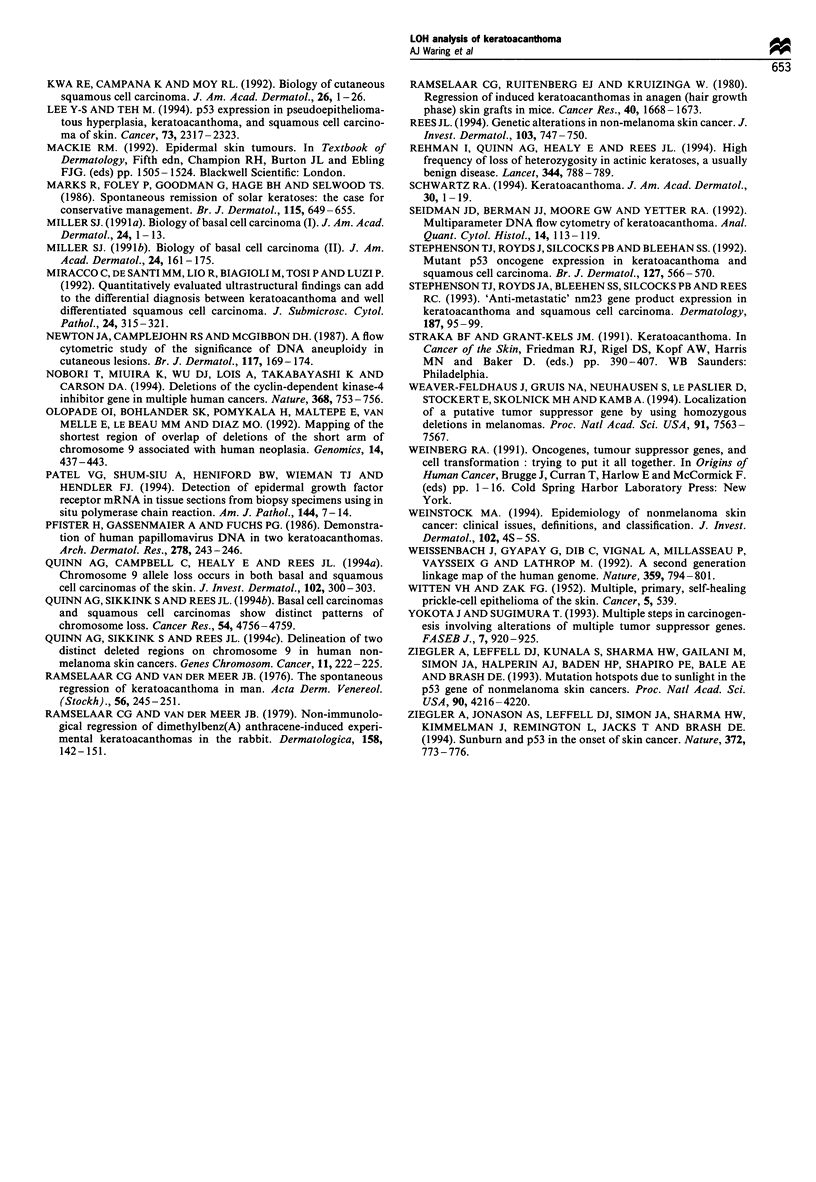# Loss of heterozygosity analysis of keratoacanthoma reveals multiple differences from cutaneous squamous cell carcinoma.

**DOI:** 10.1038/bjc.1996.113

**Published:** 1996-03

**Authors:** A. J. Waring, M. Takata, I. Rehman, J. L. Rees

**Affiliations:** Department of Dermatology, University of Newcastle upon Tyne, UK.

## Abstract

**Images:**


British Journal of Cancer (1996) 73, 649-653
© 1996 Stockton Press All rights reserved 0007-0920/96 $12.00

### Loss of heterozygosity analysis of keratoacanthoma reveals multiple differences from cutaneous squamous cell carcinoma

AJ Waring[1], M Takata[1,2], I Rehman[1] and JL Rees[1]

[1]Department of Dermatology, University of Newcastle upon Tyne, Royal Victoria Infirmary, Newcastle upon Tyne NE1 4LP, UK; [2]Department of Dermatology, Kanazawa University School of Medicine, Takaramachi 13-1, Kanazawa 920, Japan.

**Summary** Keratoacanthomas (KAs) resemble squamous cell carcinomas (SCCs) except that, unlike SCCs, after a period of rapid growth over a few months they involute completely. The basis of their regressing natural history is not known. We have examined keratoacanthomas and another benign cutaneous tumour, the basal cell papilloma (BCP), for loss of heterozygosity (LOH) at a number of loci that are frequently lost in SCCs and other skin tumours. The frequency of LOH for both KAs and BCPs was low, with only isolated losses identified at 9p, 9q and 10q in KAs [fractional allelic loss (FAL) was 1.3%], and at 9p and 17p in BCPs (FAL was 0.4%). This contrasts with previous work showing a FAL of 32% in SCC and 46% in actinic keratoses. The results show a clear difference between KA and SCC and do not support the hypothesis that KAs are SCCs that regress as a result of external (host) influences but rather suggest that KAs and SCCs are different *de novo*. LOH around the locus implicated in the multiple self-healing epitheliomata of Ferguson-Smith (9q22-q31) was shown in only 1 of 11 KAs.

**Keywords:** keratoacanthoma; benign cell papilloma; loss of heterozygosity; multiple self-healing squamous epithelioma; p53; squamous cell carcinoma

Current cellular theories of carcinogenesis emphasise the multistage nature of cancer in which there is thought to be a causal relation between the accumulation of genetic abnormalities and the clinical and biological behaviour of the tumour (Weinberg, 1991; Fearon and Vogelstein, 1990; Yokota and Sugimura, 1993). In humans the clearest example is probably the development of colorectal carcinoma, and in the mouse the development of cutaneous squamous cell carcinoma (SCC) and spindle cell carcinoma following topical application of chemical carcinogens (for reviews see Fearon and Vogelstein, 1990; Burns *et al.*, 1991). Non-melanoma skin cancer (NMSC), the commonest human malignancy in many Caucasian populations (Weinstock, 1994), offers a number of opportunities for the investigation of the relationship between genetic change and tumour phenotype. The occurrence of a range of different tumour types, all keratinocyte derived and related to ultraviolet radiation (UVR), with markedly different clinical behaviours, and in particular the presence of a number of neoplastic lesions, such as keratoacanthomas (KAs), that show spontaneous regression are of considerable experimental interest (Rees, 1994). KAs are a common keratinising squamous neoplasm, classically occurring on the UVR-exposed skin of elderly individuals, which are characterised clinically by a period of rapid growth over a 4-12 week period followed by spontaneous involution (Schwartz, 1994; Straka and Grant-Kels, 1991; Ghadially and Ghadially, 1993). Histologically KAs resemble SCCs, and although on occasions the histological differentiation from SCC may be difficult their biologically benign course allows clear differentiation (Straka and Grant-Kels, 1991; Schwartz, 1994). A number of pathogenic abnormalities have been described in KAs, including H-*ras* mutations, aneuploidy, altered p53 immunostaining and the presence of human papillomavirus (HPV) DNA (Corominas *et al.*, 1989; Newton *et al.*, 1987; Herzberg *et al.*, 1991; Kerschmann *et al.*, 1994; Stephenson *et al.*, 1992; Lee and Teh, 1994; Schwartz, 1994).

The genetic changes underlying KA are of interest for a number of reasons. Firstly, putative causal changes appear to be insufficient to allow the tumour to maintain its integrity and prolonged growth in the host; comparisons with the genetic changes found in SCC may therefore be interesting (Quinn *et al.*, 1994*b*). Secondly, a related tumour, the familial multiple self-healing squamous epithelioma (MSSE) of Ferguson-Smith, maps to chromosome 9q22-q31 (Goudie *et al.*, 1993). Although there may be histological differences between sporadic KA and MSSE, many consider the MSSE a form of familial KA (Straka and Grant-Kels, 1991; Schwartz, 1994); reports of multiple KAs occurring sporadically also suggest overlap (Witten and Zak, 1952). A final reason for interest in the genetics of KAs is the finding that another cutaneous lesion, actinic keratoses (AK) (small scaly red lesions occurring on UVR-exposed skin showing varying degrees of dysplasia), which also show a high rate of spontaneous regression (Marks *et al.*, 1986), show a frequency of allelic loss similar to, if not higher than, SCC (Rehman *et al.*, 1994). One of a number of interpretations of the allelotype data of AK is that accumulation of genetic change in skin tumours may be associated with both progression and regression (Rees, 1994; Rehman *et al.*, 1994). KAs provide a natural group of lesions on which to examine further the relation between genetic change and clinical behaviour, and we have therefore carried out loss of heterozygosity (LOH) analysis at a number of loci implicated in SCC development in a series of KAs. In order to aid interpretation and as further controls, we have carried out a similar analysis on basal cell papillomas (BCP) ('seborrhoeic keratosis'), benign lesions that are histologically characterised by thickening of the epidermis with keratinocyte immaturity, but with no known relation to either basal cell carcinoma (BCC) or SCC or any dysplasia of the skin (Mackie, 1992).

### Materials and methods

### *Clinical samples*

Twenty-four archival blocks with a diagnosis of sporadic KA and 27 blocks with a diagnosis of basal cell papilloma were retrieved for analysis from the Royal Victoria Infirmary, Newcastle upon Tyne. Clinical records and histology were reassessed by an independent clinician and dermatopathologist, with the histology being assessed without knowledge of the clinical course. Only samples that showed the typical histology of proliferating, fully developed or involutionary

Correspondence: JL Rees
Received 23 June 1995; revised 11 September 1995; accepted 29 September 1995

stages of keratoacanthoma and a clinical history of rapid growth (lesions less than 3 months old) are referred to as 'definite KAs' (Straka and Grant-Kels, 1991; Schwartz, 1994). Paraffin-embedded sections (20 $\mu$m) of tumour material (dewaxed) were microdissected from surrounding inflammatory infiltrate and normal skin on an inverted microscope. Tumour DNA and DNA from normal adjacent skin was isolated according to standard methods by proteinase K digestion and phenol–chloroform extraction (Jackson *et al.*, 1992).

### *Analysis of loss of heterozygosity*

PCR amplification of microsatellite polymorphisms was carried out with approximately 100 ng of template DNA with 200 mM deoxynucleotide triphosphates, 1 pmol of each oligonucleotide primer (Research Genetics, Huntsville, USA), one of which was end-labelled with $^{32}$P-ATP, and 1 U of *Taq* DNA polymerase (Biotaq; Bioline, London, UK) in 20 $\mu$l. Amplification consisted of 30 cycles of 1 min at 95°C, 1 min at 55°C and 1 min at 72°C with a final 10 min extension time of 72°C. Loading buffer (10 $\mu$l) was added at the end of each reaction and samples were heat denatured and electrophoresed through 6% denaturing polyacrylamide gels. Gels were fixed in 10% acetic acid–10% methanol, vacuum dried and exposed to Fuji XR film for up to 24 h. Allelic loss was scored without knowledge of the putative tumour type and independently of the re-reviewed histological and clinical details. A significant reduction in the signal intensity of one of the two tumour alleles was recorded as LOH (although this does not formally exclude allelic imbalance secondary to amplification of one allele).

LOH analysis was carried out for loci that previous studies have shown to be frequently lost in SCC and AK (3p, 9p, 13q, 17p and 17q) (Quinn *et al.*, 1994*b*; Rehman *et al.*, 1994), and on other arms chosen at random (see Table I). Loci of markers are referred to as described by Weissenbach *et al.* (1992). The frequency of LOH at a locus is given by the number of losses at the locus divided by the number of tumours informative for that locus. The fractional allelic loss (FAL) is given by summing the LOH score at all loci and dividing by the number of tumours examined that were informative at the loci. In view of the mapping of two familial skin cancer syndromes [naevoid basal cell carcinoma syndrome (NBCCS) and MSSE] to 9q22–q31 (Farndon *et al.*, 1992; Gailani *et al.*, 1992; Goudie *et al.*, 1993), and evidence from LOH studies of a putative tumour-suppressor gene on 9p for cutaneous SCC (Quinn *et al.*, 1994*a*, *b*, *c*), a more detailed analysis using multiple markers on chromosome 9 was carried out on the keratoacanthomas.

**Table I** Frequency of loss of microsatellite markers used for LOH analysis of 11 keratoacanthomas (KAs) and 27 basal cell papillomas (BCPs); numbers of tumours showing loss/number of informative cases at each chromosome arm. Markers are described by Weissenbach *et al.* (1992)

| *Chromosome* | *Locus* | *Definite KAs* | *Probable KAs* | *BCPs* |
|---|---|---|---|---|
| 1p | *D1S201* | 0/8 | 0/9 | 0/16 |
| 1q | *D1S212* | 0/10 | 0/11 | 0/17 |
| 2p | *D2S149* | 0/7 | 0/10 | 0/24 |
| 2q | *D2S163* | 0/10 | 0/10 | 0/21 |
| 3p | *D3S1293* | 0/9 | 2/11 | 0/23 |
| 3q | *D3S1268* | 0/9 | 0/7 | 0/22 |
| 4p | *D4S394* | 0/9 | 0/8 | 0/12 |
| 4q | *D4S402* | 0/8 | 0/8 | 0/19 |
| 5p | *D5S419* | 0/10 | 0/9 | 0/19 |
| 5q | *D5S410* | 0/8 | 0/6 | 0/22 |
| 6p | *D6S299* | 0/8 | 0/9 | 0/23 |
| 6q | *D6S262* | 0/8 | 0/11 | 0/23 |
| 7p | *D7S481* | 0/8 | 0/7 | 0/9 |
| 9p | *D9S171* | 1/7 | 1/8 | 0/19 |
| 9q | *D9S160* | 1/9 | 0/9 | 1/16 |
| 10q | *D10S185* | 1/7 | 0/7 | ND |
| 11p | *D11S922* | 0/11 | 0/10 | 0/27 |
| 11q | *D11S910* | 0/5 | 0/7 | ND |
| 12q | *D12S86* | 0/11 | 0/9 | 0/25 |
| 13q | *D13S170* | 0/9 | 0/10 | 0/14 |
| 17p | *D17S796* | 0/10 | 1/7 | 1/19 |
| 17q | *D17S785* | 0/8 | 1/7 | 0/19 |
| 18p | *D18S59* | 0/11 | 0/8 | 0/21 |
| 18q | *D18S70* | 0/9 | 0/10 | 0/21 |
| 21q | *D21S262* | 0/8 | 0/4 | ND |
| 22q | *D22S283* | 0/11 | 0/9 | 0/22 |

ND, no data.

### Results

### *Tumour sample diagnosis*

Review of the histology and clinical details confirmed the original diagnosis in all the basal cell papillomas. However adoption of strict criteria relying on both characteristic clinical history *and* histopathological assessment for the diagnosis of KA showed that in only 11 of the 24 putative tumours could a definite diagnosis of KA be sustained for the purposes of this study (referred to as 'definite KAs'). Of the remaining samples, one block contained only normal tissue, another was clearly a SCC, and the histology or the clinical history in the other cases was not classical, although the likely diagnosis was thought to be KA. Where tissue was available, and with the exception of the definite SCC, these samples were analysed and are referred to as 'probable KAs' ($n=11$).

### *LOH in keratoacanthomas and basal cell papillomas*

The frequency of LOH for both KAs and benign papillomas was low with only isolated losses identified (Table I). Of the 11 'definite KAs' only two tumours showed LOH occurring at a total of three loci; one KA showed allelic loss on chromosome arms 9p (*D9S162*) and 10q (*D10S185*), one showed allelic loss on chromosome arm 9q (*D9S160*) (Figure 1), and the other nine KAs showed no LOH at any of the chromosome arms analysed. The FAL based on the 26 loci examined was 1.3% (3/228). Of the 27 BCPs studied LOH was identified in only two tumours; one showed LOH at 9q (*D9S160*) and the other at 17p (*D17S796*) (Figure 2). The FAL based on the 23 loci examined was 0.4% (2/453).

Analysis of the 'probable KAs' (those that did not meet strict histological and clinical criteria) was also carried out. These 11 lesions showed a low frequency of loss with a FAL score based on the 26 loci examined of 2.2% (5/222) with LOH occurring at 3p (2/11), 9p (1/8), 17p (1/7) and 17q (1/7). By contrast, the one lesion recognised as a SCC showed LOH at 7 of 20 informative chromosome markers (3p, 3q, 9p, 9q, 10q, 17p and 18p).

### *Deletion mapping of chromosome 9 in the definite keratoacanthomas*

The pattern of chromosome 9 loss was examined in more detail in all 11 definite KAs using ten microsatellite markers. One KA showed loss of distal 9p markers *D9S162* and *D9S171* with retention of 9p markers centromeric to *D9S171* and all informative 9q markers examined (*D9S169*, *D9S166*, *D9S167*, *D9S152*, *D9S176* and *D9S160*). The area of loss in this lesion encompassed the interferon cluster at 9p21–q22 and the region containing the genes coding for p16 and p15 (Olopade *et al.*, 1992; Kamb *et al.*, 1994; Nobori *et al.*, 1994; Weaver-Feldhaus *et al.*, 1994). One other KA showed loss of 9q markers *D9S160* and *D9S176*. However, attempts to further define the extent of loss of chromosome 9 in this lesion were impossible because analysis with other proximal 9q markers and 9p markers (*D9S180*, *D9S152*, *D9S167* and *D9S166*, *D9S165*, *D9S169*, *D9S171* and *D9S162*) was either uninformative or gave no PCR product. The area of loss in this tumour encompassed the locus or loci underlying the NBCCS and the MSSE syndrome at 9q22–q31 (Gailani *et al.*, 1992; Farndon *et al.*, 1992; Goudie *et al.*, 1993).

### Discussion

There are many similarities between KAs and SCCs; for any one tumour, no single investigation can distinguish them reliably (Straka and Grant-Kels, 1991; Mackie, 1992; Schwartz, 1994). Both types of lesions may share a similar clinical and histopathological appearance; both occur predominantly on sun-exposed skin and are more common in patients receiving immunosuppressive therapy; both KAs and SCCs can be induced by the same chemical carcinogen protocols in animals; and both have been reported to be associated with HPV infection (although whether this plays a causal role is unknown) (Gassenmaier *et al*., 1986; Pfister *et al*., 1986; Kwa *et al*., 1992; Schwartz, 1994). Studies assessing DNA ploidy levels, nuclear morphometry, p53 immunostaining, nm23 expression and a variety of morphological criteria have all shown overlap between KAs and SCCs (Herzberg *et al*., 1991; Miracco *et al*., 1992; Seidman *et al*., 1992; Stephenson *et al*., 1992, 1993; Helander *et al*., 1993). The only consistent difference lies in the characteristic clinical natural history of rapid growth followed by spontaneous involution of the KA compared with the persistence and possible metastasis of the SCC (Kwa *et al*., 1992).

A number of arguments have been advanced to explain the natural history of KAs, including that they are follicular tumours with the period of growth and involution reflecting the normal hair cycle periods of anagen and catagen; that involution is immunologically mediated; and that KAs are associated with genetic events (including *ras* mutations) that lead to regression rather than progression (Ramselaar and van der Meer, 1976, 1979; Ramselaar *et al*., 1980; Corominas *et al*., 1989; Straka and Grant-Kels, 1991; Patel *et al*., 1994; Schwartz, 1994). There is support for and against each of these hypotheses and none of these explanations of KA involution is mutually exclusive.

The majority of KAs in the present study showed no LOH at any of the markers studied whereas LOH is common on chromosome arms 3p, 9p, 9q, 13q, 17p and 17q in SCCs and AKs. The FAL score for the KAs ('definite KAs') at these six loci of 4% (2/50) contrasts with the corresponding FAL score of 32% for the SCCs and 46% for the AKs (Quinn *et al*., 1994*b*; Rehman *et al*., 1994). The results are also different from the 59% LOH seen on 9q for basal cell carcinomas (Quinn *et al*., 1994*b*). The inclusion of only lesions that had both typical histological features and a characteristic clinical history in the KA group make it unlikely that the results are due to misdiagnosis of KAs as SCCs. The fact that a similar FAL score was seen in the 'probable KA' group is reassuring in this respect. Our previous findings showing a high frequency of LOH on chromosome 9 in basal cell

**Figure 1** (**a**) Representative autoradiograph showing allele loss in keratoacanthoma and squamous cell carcinoma at *D9S162* (chromosome arm 9p). Lanes 1–8 show PCR product from normal (N) and tumour (T) DNA from four patients with KAs. Lanes 9–10 show PCR product from normal and tumour DNA from one patient with SCC. One KA in lane 6 and the SCC in lane 10 show a diminished or absent band indicating LOH at *D9S162*. (**b**) Representative autoradiograph showing allele loss in keratoacanthoma at *D10S185* (chromosome arm 10q). Lanes 1–6 show PCR product from normal and tumour DNA from three patients with KAs. One KA in lane 4 shows LOH at *D10S185*.

**Figure 2** Representative autoradiograph showing allele loss in basal cell papillomas at *D9S160* (chromosome arm 9q). Lanes 1–9 show PCR product from normal (N) and tumour (T) DNA from four patients with BCPs. Lanes 8 and 9 show analysis of tumour DNA from one patient with two BCPs; one of these BCPs in lane 9 shows LOH at *D9S160*.

carcinomas (Quinn *et al.*, 1994*b*), and a high FAL in AKs which are much smaller lesions than KAs (Rehman *et al.*, 1994), also argues against technical failure owing to an inability to separate tumour from stroma.

The rarity of LOH in KAs has bearings on their pathogenesis. Firstly it suggests that attempts to explain the involution of KAs on the basis that they are really SCCs that are for some other reason subsequently attacked by the immune system may be mistaken (Patel *et al.*, 1994). Secondly the failure to find LOH around the locus responsible for the MSSE syndrome is worthy of comment. There are differences of opinion about the nature of the tumours seen in MSSE with many authors referring to them as KAs, whereas others argue that they are a specific form of SCC that undergoes involution (Straka and Grant-Kels, 1991; Mackie, 1992; Ghadially and Ghadially, 1993; Schwartz, 1994). The finding of LOH in only 1 of 11 sporadic KAs at loci close to the MSSE/naevoid basal cell carcinoma syndrome at 9q22–q31 raises the possibility that the tumours are not equivalent genetically. However, in the absence of studies showing LOH in MSSE lesions, the comparison may not be valid as not all familial cancer susceptibility genes are accompanied by LOH: we cannot therefore exclude the same gene's involvement in sporadic KA and MSSE. The infrequent LOH on chromosome 9 is also of interest in the light of theories suggesting that KAs are follicular (or more strictly appendageal) in origin. Several lines of evidence suggest that BCC are derived from follicular cell progenitors (Miller, 1991*a*, *b*), and hence the absence of LOH on chromosome 9 argues that if KA are follicular then they have a different pathogenesis from BCC.

LOH on chromosome 17 was seldom seen in the KAs, whereas loss involving a number of markers on chromosome 17 is commonly seen in other forms of NMSC including AKs, Bowen's disease and SCC (Rehman *et al.*, 1994; Ziegler *et al.*, 1994), as is the presence of p53 mutation (Campbell *et al.*, 1993*b*; Brash *et al.*, 1991; Ziegler *et al.*, 1994). This suggests a further difference between KA and SCCs, although it is not possible to exclude a role for p53 in KA as other methods of inactivating p53 may occur in KA; for instance mutations on both alleles of p53 as has been reported in BCC (Campbell *et al.*, 1993*a*; Ziegler *et al.*, 1993), small deletions, or interaction with proteins that may target p53 degradation or interfere in the p53 pathway all of which could have been missed. Other studies have however failed to find p53 mutations in KAs (Kubo *et al.*, 1994).

The finding of occasional LOH in the BCPs and two of the KAs is open to a number of interpretations besides the obvious one that these changes are causally related to the pathogenesis of these lesions. Evidence of LOH does however suggest that these lesions are clonal. The fact that the two KAs showing LOH showed LOH on chromosome 9, and the involvement of loci on chromosome 9 in other forms of skin cancer, might be interpreted as support for these abnormalities being causally important in KA pathogenesis. Similarly in the 'probable KA' group LOH occurred at loci known to be lost at high frequency in SCC and AK, namely chromosome arms 3p, 9p, 17p and 17q. However, inevitably, interpretation of the probable KA results is difficult, as although most of the lesions are likely to represent KA, squamous cell carcinoma in one or more of the samples cannot be excluded, and the presence of SCC would clearly bias the results towards loss of those areas that occur in SCC development. An alternative, and perhaps less likely, hypothesis is that the LOH may be unrelated to the development of the lesion but merely reflects background LOH in the proliferative compartment in skin.

In summary the LOH data show a clear difference between KAs and SCCs. The interpretation of KAs as *de novo* squamous cell carcinomas which for some other reason involute seems unlikely, rather the results suggest that from early in their pathogenesis KAs are distinct from SCC. The low level of LOH in KAs contrasts with the predominantly isolated loss on chromosome 9 that occurs in basal cell carcinoma and the high frequency of LOH in lesions such as actinic keratoses that are also keratinocyte-derived and show a high rate of regression.

**Acknowledgements**
This work was supported by the North of England Cancer Research Campaign (NECRC). IR is a NECRC PhD student.

### References

BRASH DE, RUDOLPH JA, SIMON JA, LIN A, MCKENNA GJ, BADEN HP, HALPERIN AJ AND PONTEN J. (1991). A role for sunlight in skin cancer: UV-induced p53 mutations in squamous cell carcinoma. *Proc. Natl Acad. Sci. USA*, **88**, 10124–10128.

BURNS PA, BREMNER R AND BALMAIN A. (1991). Genetic changes during mouse skin tumorigenesis. *Environ. Health Perspect.*, **93**, 41–44.

CAMPBELL C, QUINN AG, ANGUS B AND REES JL. (1993*a*). The relation between p53 mutation and p53 immunostaining in non-melanoma skin cancer. *Br. J. Dermatol.*, **129**, 235–241.

CAMPBELL C, QUINN AG, RO YS, ANGUS B AND REES JL. (1993*b*). p53 mutations are common and early events that precede tumor invasion in squamous cell neoplasia of the skin. *J. Invest. Dermatol.*, **100**, 746–748.

COROMINAS M, KAMINO H, LEON J AND PELLICER A. (1989). Oncogene activation in human benign tumors of the skin (keratoacanthomas) : is H-ras involved in differentiation as well as proliferation. *Proc. Natl Acad. Sci. USA*, **86**, 6372–6376.

FARNDON PA, DEL MASTRO RG, EVANS DGR AND KILPATRICK MW. (1992). Location of gene for Gorlin syndrome. *Lancet*, **339**, 581–582.

FEARON ER AND VOGELSTEIN B. (1990). A genetic model for colorectal tumorigenesis. *Cell*, **61**, 759–767.

GAILANI MR, BALE SJ, LEFFEL DJ, DIGIOVANNA JJ, PECK GL, POLLAK S, DRUM MA, PASTAKIA B, MCBRIDE OW, KASE R, GREENE M, MULVIHILL JJ AND BALE AE. (1992). Developmental defects in Gorlin's syndrome related to a putative tumor suppressor gene on chromosome 9. *Cell*, **69**, 111–117.

GASSENMAIER A, PFISTER H AND HORNSTEIN OP. (1986). Human papillomavirus 25-related DNA in solitary keratoacanthoma. *Arch. Dermatol. Res.*, **279**, 73–76.

GHADIALLY R AND GHADIALLY FN. (1993). Keratoacanthoma. In *Dermatology in General Medicine*, Fitzpatrick TB, Eisen AZ, Wolff K, Freedberg IM and Austen KF. (eds) pp. 848–855. McGraw-Hill: New York.

GOUDIE DR, YUILLE MAR, LEVERSHA MA, FURLONG RA, CARTER NP, LUSH MJ, AFFARA NA AND FERGUSON-SMITH MA. (1993). Multiple self healing squamous epitheliomata (ESS1) mapped to chromosome 9q22-q31 in families with common ancestry. *Nat. Gen.*, **3**, 165–169.

HELANDER SD, PETERS MS AND PITTELKOW MR. (1993). Expression of p53 protein in benign and malignant epidermal pathologic conditions. *J. Am. Acad. Dermatol.*, **29**, 741–748.

HERZBERG AJ, KERNS BJ, POLLACK V AND KINNEY RB. (1991). DNA image cytometry of keratoacanthoma and squamous cell carcinoma. *J. Invest. Dermatol.*, **97**, 495–500.

JACKSON DP, HAYDEN JD AND QUIRKE P. (1992). Extraction of nucleic acid from fresh and archival material. In *PCR, a Practical Approach*, McPherson MJ, Quirke P and Taylor GR. (eds) pp. 29–50. Oxford University Press: Oxford.

KAMB A, NELLEKE AG, WEAVER-FELDHAUS J, LIU Q, HARSHMAN K, TAVTIGIAN SV, STOCKERKT E, DAY RS, JOHNSON BE AND SKOLNICK MH. (1994). A cell cycle regulator potentially involved in genesis of many tumor types. *Science*, **264**, 436–440.

KERSCHMANN RL, MCCALMONT TH AND LEBOIT PE. (1994). p53 oncoprotein expression and proliferation index in keratoacanthoma and squamous cell carcinoma. *Arch. Dermatol.*, **130**, 181–185.

KUBO Y, URANO Y, YOSHIMOTO K, IWAHANA H, FUKUHARA K, ARASE S AND ITAKURA M. (1994). p53 gene mutations in human skin cancers and precancerous lesions: comparison with immunohistochemical analysis. *J. Invest. Dermatol.*, **102**, 440–444.

KWA RE, CAMPANA K AND MOY RL. (1992). Biology of cutaneous squamous cell carcinoma. *J. Am. Acad. Dermatol.*, **26**, 1–26.

LEE Y-S AND TEH M. (1994). p53 expression in pseudoepitheliomatous hyperplasia, keratoacanthoma, and squamous cell carcinoma of skin. *Cancer*, **73**, 2317–2323.

MACKIE RM. (1992). Epidermal skin tumours. In *Textbook of Dermatology*, Fifth edn, Champion RH, Burton JL and Ebling FJG. (eds) pp. 1505–1524. Blackwell Scientific: London.

MARKS R, FOLEY P, GOODMAN G, HAGE BH AND SELWOOD TS. (1986). Spontaneous remission of solar keratoses: the case for conservative management. *Br. J. Dermatol.*, **115**, 649–655.

MILLER SJ. (1991*a*). Biology of basal cell carcinoma (I). *J. Am. Acad. Dermatol.*, **24**, 1–13.

MILLER SJ. (1991*b*). Biology of basal cell carcinoma (II). *J. Am. Acad. Dermatol.*, **24**, 161–175.

MIRACCO C, DE SANTI MM, LIO R, BIAGIOLI M, TOSI P AND LUZI P. (1992). Quantitatively evaluated ultrastructural findings can add to the differential diagnosis between keratoacanthoma and well differentiated squamous cell carcinoma. *J. Submicrosc. Cytol. Pathol.*, **24**, 315–321.

NEWTON JA, CAMPLEJOHN RS AND MCGIBBON DH. (1987). A flow cytometric study of the significance of DNA aneuploidy in cutaneous lesions. *Br. J. Dermatol.*, **117**, 169–174.

NOBORI T, MIUIRA K, WU DJ, LOIS A, TAKABAYASHI K AND CARSON DA. (1994). Deletions of the cyclin-dependent kinase-4 inhibitor gene in multiple human cancers. *Nature*, **368**, 753–756.

OLOPADE OI, BOHLANDER SK, POMYKALA H, MALTEPE E, VAN MELLE E, LE BEAU MM AND DIAZ MO. (1992). Mapping of the shortest region of overlap of deletions of the short arm of chromosome 9 associated with human neoplasia. *Genomics*, **14**, 437–443.

PATEL VG, SHUM-SIU A, HENIFORD BW, WIEMAN TJ AND HENDLER FJ. (1994). Detection of epidermal growth factor receptor mRNA in tissue sections from biopsy specimens using in situ polymerase chain reaction. *Am. J. Pathol.*, **144**, 7–14.

PFISTER H, GASSENMAIER A AND FUCHS PG. (1986). Demonstration of human papillomavirus DNA in two keratoacanthomas. *Arch. Dermatol. Res.*, **278**, 243–246.

QUINN AG, CAMPBELL C, HEALY E AND REES JL. (1994*a*). Chromosome 9 allele loss occurs in both basal and squamous cell carcinomas of the skin. *J. Invest. Dermatol.*, **102**, 300–303.

QUINN AG, SIKKINK S AND REES JL. (1994*b*). Basal cell carcinomas and squamous cell carcinomas show distinct patterns of chromosome loss. *Cancer Res.*, **54**, 4756–4759.

QUINN AG, SIKKINK S AND REES JL. (1994*c*). Delineation of two distinct deleted regions on chromosome 9 in human non-melanoma skin cancers. *Genes Chromosom. Cancer*, **11**, 222–225.

RAMSELAAR CG AND VAN DER MEER JB. (1976). The spontaneous regression of keratoacanthoma in man. *Acta Derm. Venereol. (Stockh).*, **56**, 245–251.

RAMSELAAR CG AND VAN DER MEER JB. (1979). Non-immunological regression of dimethylbenz(A) anthracene-induced experimental keratoacanthomas in the rabbit. *Dermatologica*, **158**, 142–151.

RAMSELAAR CG, RUITENBERG EJ AND KRUIZINGA W. (1980). Regression of induced keratoacanthomas in anagen (hair growth phase) skin grafts in mice. *Cancer Res.*, **40**, 1668–1673.

REES JL. (1994). Genetic alterations in non-melanoma skin cancer. *J. Invest. Dermatol.*, **103**, 747–750.

REHMAN I, QUINN AG, HEALY E AND REES JL. (1994). High frequency of loss of heterozygosity in actinic keratoses, a usually benign disease. *Lancet*, **344**, 788–789.

SCHWARTZ RA. (1994). Keratoacanthoma. *J. Am. Acad. Dermatol.*, **30**, 1–19.

SEIDMAN JD, BERMAN JJ, MOORE GW AND YETTER RA. (1992). Multiparameter DNA flow cytometry of keratoacanthoma. *Anal. Quant. Cytol. Histol.*, **14**, 113–119.

STEPHENSON TJ, ROYDS J, SILCOCKS PB AND BLEEHAN SS. (1992). Mutant p53 oncogene expression in keratoacanthoma and squamous cell carcinoma. *Br. J. Dermatol.*, **127**, 566–570.

STEPHENSON TJ, ROYDS JA, BLEEHEN SS, SILCOCKS PB AND REES RC. (1993). 'Anti-metastatic' nm23 gene product expression in keratoacanthoma and squamous cell carcinoma. *Dermatology*, **187**, 95–99.

STRAKA BF AND GRANT-KELS JM. (1991). Keratoacanthoma. In *Cancer of the Skin*, Friedman RJ, Rigel DS, Kopf AW, Harris MN and Baker D. (eds.) pp. 390–407. WB Saunders: Philadelphia.

WEAVER-FELDHAUS J, GRUIS NA, NEUHAUSEN S, LE PASLIER D, STOCKERT E, SKOLNICK MH AND KAMB A. (1994). Localization of a putative tumor suppressor gene by using homozygous deletions in melanomas. *Proc. Natl Acad. Sci. USA*, **91**, 7563–7567.

WEINBERG RA. (1991). Oncogenes, tumour suppressor genes, and cell transformation : trying to put it all together. In *Origins of Human Cancer*, Brugge J, Curran T, Harlow E and McCormick F. (eds) pp. 1–16. Cold Spring Harbor Laboratory Press: New York.

WEINSTOCK MA. (1994). Epidemiology of nonmelanoma skin cancer: clinical issues, definitions, and classification. *J. Invest. Dermatol.*, **102**, 4S–5S.

WEISSENBACH J, GYAPAY G, DIB C, VIGNAL A, MILLASSEAU P, VAYSSEIX G AND LATHROP M. (1992). A second generation linkage map of the human genome. *Nature*, **359**, 794–801.

WITTEN VH AND ZAK FG. (1952). Multiple, primary, self-healing prickle-cell epithelioma of the skin. *Cancer*, **5**, 539.

YOKOTA J AND SUGIMURA T. (1993). Multiple steps in carcinogenesis involving alterations of multiple tumor suppressor genes. *FASEB J.*, **7**, 920–925.

ZIEGLER A, LEFFELL DJ, KUNALA S, SHARMA HW, GAILANI M, SIMON JA, HALPERIN AJ, BADEN HP, SHAPIRO PE, BALE AE AND BRASH DE. (1993). Mutation hotspots due to sunlight in the p53 gene of nonmelanoma skin cancers. *Proc. Natl Acad. Sci. USA*, **90**, 4216–4220.

ZIEGLER A, JONASON AS, LEFFELL DJ, SIMON JA, SHARMA HW, KIMMELMAN J, REMINGTON L, JACKS T AND BRASH DE. (1994). Sunburn and p53 in the onset of skin cancer. *Nature*, **372**, 773–776.